# Antiherpetic Effects of *Gynura procumbens*

**DOI:** 10.1155/2013/394865

**Published:** 2013-09-15

**Authors:** Siripen Jarikasem, Somyot Charuwichitratana, Sontana Siritantikorn, Wasan Chantratita, Magdy Iskander, August Wilhelm Frahm, Weena Jiratchariyakul

**Affiliations:** ^1^Department of Pharmacognosy, Faculty of Pharmacy, Mahidol University, 447 Sri Ayudhya Road, Rajathevi District, Bangkok 10400, Thailand; ^2^Pharmaceutical and Natural Products Department, Thailand Institute of Scientific and Technological Research, Pathum Thani 12120, Thailand; ^3^Division of Dermatology, Department of Medicine, Faculty of Medicine, Ramathibodi Hospital, Mahidol University, Rama VI Road, Bangkok 10400, Thailand; ^4^Department of Microbiology, Faculty of Medicine Siriraj Hospital, Prannok Road, Bangkok Noi District, Bangkok 10700, Thailand; ^5^Department of Pathology, Faculty of Medicine Ramathibodi Hospital, Mahidol University, Rama VI Road, Bangkok 10400, Thailand; ^6^Department of Medicinal Chemistry, Victorian College of Pharmacy, Monash University, Melbourne, VIC 3052, Australia; ^7^Department of Pharmaceutical and Medicinal Chemistry, Institute of Pharmaceutical Sciences, Freiburg University, 79104 Freiburg, Germany

## Abstract

The ethanol extract of *Gynura procumbens* showed virucidal and antireplicative actions against herpes simplex virus HSV-1 and HSV-2. It was further chromatographed on MCI gel CHP20P column giving the extract fractions F1 (water), F2 (water-methanol) F3 (methanol), and F4 (ethyl acetate). All but F1 had virucidal action against both viral types. We reported here the active compounds from F2 and F3. The antiherpetic compounds of F2 was a mixture of dicaffeoylquinic acids with virucidal and antireplicative actions against HSV-2 (IC_50_ 96.0 and 61.0 **μ**g/mL, resp.) Virucidal compounds of F3 were a mixture of **β**-sitosterol and stigmasterol (IC_50_ 250.0 **μ**g/mL against HSV-1), a mixture of **β**-sitosteryl and stigmasteryl glucosides (IC_50_ 50.0 **μ**g/mL against HSV-2) and 1, 2-bis-dodecanoyl-3-**α**-D-glucopyranosyl-*sn*-glycerol (IC_50_ of 40.0 **μ**g/mL against HSV-2). Herbal products containing 1 and 2% of standardized ethanol extract were prepared. Double-blind randomized controlled clinical trial of the products was performed in patients with recurrent herpes labialis. Results showed that the number of patients, whose lesions healed within 7 days and the average healing time of both groups differed insignificantly. Viral culture on D7 indicated a decrease of infected patients from 48.7% to 7.69% in treated group whereas in placebo group the infected patients decreased from 31.25% to 20.00%. The viral reduction in treated group indicated the benefit of the product. Insignificant result might arise from a low number of participated patients and insufficient concentration of plant extract in herbal product.

## 1. Introduction

*Gynura procumbens* (Lour.) Merr. is mentioned in traditional Chinese medicine as a topical anti-inflammatory remedy [1]. In Southeast Asia, *Gynura* plants are widely distributed. In Thailand, *G. pseudochina* var. *hispida* (Thai name: Wan Mahakaan) is externally used as anti-itching, anti-inflammatory, and antiherpes virus [2]. In Singapore, Malaysia, and Indonesia, the plant has been traditionally used as remedies for eruptive fever, rash, kidney disease, migraine, constipation, hypertension, diabetes mellitus, and cancer [3]. The ethanol extract from the leaf reduced the mouse ear oedema induced by croton oil [4]. Phytochemical studies of *Gynura* plants resulted in the discovery of pyrrolizidine alkaloids [5–7], spirostanol [8], coumarins [7–9], and anthocyanins [10]. Since *G. procumbens* so far has not been explored towards antiherpes viral activity, on the other hand, according to traditional Thai medicine, the utilization of the plant may be associated with herpes viral infection; the investigation of the plant components for the antiherpetic activity is performed.

## 2. Materials and Methods

*Plant Material*. *Gynura procumbens* was collected from Chanthaburi province, Thailand, in October 1994. The plant was identified by Ms. Leena Phupatpong, an expert botanist at the Forest Herbarium (BKF), Royal Forest Department, Ministry of Agriculture and Cooperatives, Bangkok, Thailand. A voucher specimen (BKF no. 127362) was deposited at the same place. 

*Extraction and Isolation*. The aerial parts of the plant (25 kg, fresh) were washed, cut into pieces, dried in a hot-air oven (60°C), and ground, yielding 1.7 kg of the coarse-powdered drug. It was successively extracted in a Soxhlet apparatus using petroleum ether (40–60°C), dichloromethane, and ethanol. The solvents were removed under reduced pressure. The dry ethanol extract (114 g) was further separated using chromatographic columns with different packing materials. MCI gel CHP20P column fractionated the extract into the fractions F1 (water), F2 (water-methanol 1 : 1), F3 (methanol), and F4 (ethyl acetate). F2, F3, and F4 were active against herpes virus. 

**Figure 1:**
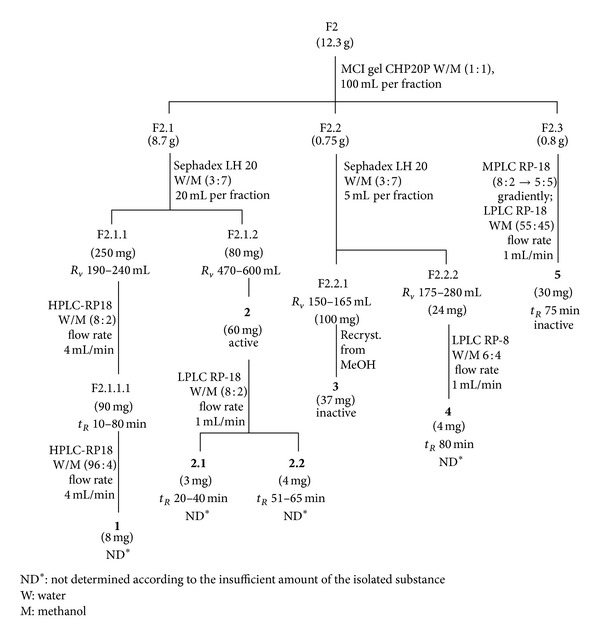
Isolation of components **1**–**5** from F2.

**Figure 2:**
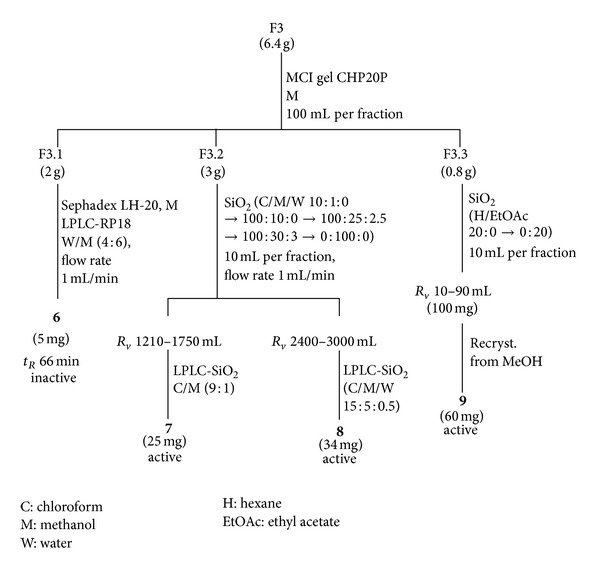
Isolation of components **6**–**9** from F3.

F2 (12.3 g) and F3 (6.4 g) were progressively chromatographed on MCI gel CHP20P using aqueous methanol (1 : 1) for F2 and methanol for F3 as solvent systems, resulting in F2.1 (8.7 g), F2.2 (0.75 g), and F2.3 (0.8 g) and F3.1 (2.0 g), F3.2 (3.0 g), and F3.3 (0.8 g), respectively. Compounds **1**, **2**, **3**, **4**, and **5** were isolated from F2 (Figure 1), while compounds **6**, **7**, **8**, and **9** were isolated from F3 (Figure 2). F4 was not further studied. 

*Cell Line*. The Vero cell line (African green monkey kidney cells) was grown and maintained in Eagle's minimum essential medium (MEM) supplemented with 10% fetal calf serum and antibiotics (5 × 10^4^ cells per well). 

*Virus*. HSV-1, strain KOS, and HSV-2, strain Baylor 186, were obtained from the Department of Microbiology, Faculty of Medicine Siriraj Hospital, Mahidol University, Bangkok, Thailand. The quantity of 100 plaque forming unit (PFU) per mL were used for experiments. 

*Evaluation of Cytotoxicity*. Serial 2-fold dilutions of the test sample in the maintenance medium were added to Vero monolayer. After incubation at 37°C for 5 days, cytotoxicity was determined by vital staining with 1% crystal violet in 10% formalin for 30 min. The highest concentration of the test sample which did not exhibit cytotoxicity represented the maximum nontoxic dose (MNTD). Serial 2-fold dilutions of MNTD were used to perform the antiviral assay. 

*Antiviral Assay*. Antiviral activity was determined by plaque reduction assay on confluent Vero cells growing in 96-well tissue culture plates. The test included three treatments, that is, inactivation and pre- and posttreatments.

Inactivation (To determine the neutralizing activity of the test sample against virus infectivity (virucidal action)): virus (100 *μ*L) was incubated with test sample (100 *μ*L) at 37°C for 1 h. The mixture was added in duplicated wells of monolayer cells and incubated at 37°C for 1 h. After washing the cells, MEM and semisolid media (0.4% gum tragacanth) were added to the cultures, which were then incubated at 37°C for 3 days, and stained with crystal violet.

Pre-treatment (to determine the inhibitory activity of viral adsorption or penetration): test sample (100 *μ*L) was added to duplicated wells of monolayer cells and incubated at 37°C for 24 h. After washing the culture, cells were infected with virus (100 *μ*L) and incubated at 37°C for 1 h. Semisolid media were added to the duplicated wells of monolayer cells after washing out the non adsorbed virus. The cultures were incubated at 37°C for 3 days and then stained with crystal violet.

Post-treatment (to determine the inhibitory activity of intracellular viral replication): the monolayer cells were infected with virus and incubated at 37°C for 1 h. After washing the cells, the test sample (100 *μ*L) and semisolid media were added in duplicated wells, incubated at 37°C for 3 days and then stained with crystal violet. 

*Control*. Cell control: monolayer cell in MEM and semisolid media. Acyclovir was used as the positive control. Virus control: monolayer cells infected with 100 PFU/mL of virus, MEM, and semisolid media. 

Calculation of plaque inhibitory capacity: percent of plaque reduction compared to the culture without treatment (virus control) was calculated. The 50% inhibition concentration (IC_50_) of the active substance or sample was determined as the lowest concentration which reduced plaque numbers by 50% in treated compared to untreated cultures.

The samples were dissolved in dimethyl sulfoxide (DMSO) and diluted with the maintenance medium. The final concentration of DMSO in the test sample was 0.3%.

*Viral Culture*. Vero cells were grown in growth medium, MEM (Earle's salt, JR Scientific, Inc., Woodland, CA, USA) supplemented with 10% fetal bovine serum (Gibco, Grand Island, USA) and 100 unit/mL penicillin G, and 100 *μ*g/mL streptomycin (M&H Manufacturing Co., Ltd., Thailand), and 0.01 M HEPES (N-2-hydroxyethyl-piperazine-N′-2-ethane sulfonic acid) (Gibco, Grand Island, USA). Maintenance media were prepared as growth media except the concentration of fetal bovine serum was reduced to 2%. 

Vero cell monolayers in culture flask were dispersed by using 0.25% (1x) trypsin (JR Scientific, Inc., Woodland, CA, USA). It was performed after removing the growth media and washing twice with 5 mL PBS. After discarding PBS, one mL of trypsin was added and cells were incubated at 37°C for 2–5 min; then the culture flask was gently shaken until the cells were detached and 5 mL of the growth media was added. These cells were counted to 10^5^ cells/mL and 1.5 mL of cells were added into the culture tube containing cover glass and then incubated at 37°C in 5% CO_2_ overnight. After the incubation, the confluent Vero cells were inoculated with 0.1 multiplicity of infection (MOI) of HSV-1 and HSV-2 viruses (100 *μ*L/culture tube) (Nunclon surface, Nunc Brand product, Denmark) as positive control. The culture was incubated at 37°C in 5% CO_2_ for 1 h with shaking at 15 min interval time before adding the maintenance media. The sample from vesicle swab collected in virus transport media (VTM) was processed by refrigerated centrifugation at 10,000 rpm for 10 min. Then, 100 *μ*L of the supernatant was inoculated in 10^5^ cells/mL confluent Vero cells of culture tube containing cover glass and incubated virus at 37°C in 5% CO_2_ for 1 h with shaking at 15 min interval time before adding the maintenance media. The confluent Vero cells of 10^5^ cells/mL per culture tube added with maintenance media were used as negative control. Each experiment was carried out in duplicate tubes. All culture tubes of samples, positive and negative control were incubated and examined for cytopathic effect (CPE). The Vero cells on the cover glass were determined for HSV-infected cells and the supernatants were kept at −70°C in order to subpassage culture again. The Vero cells on the cover glass were fixed with chilled acetone for 10 min and air-dried. The detection of HSV in Vero cells using a specific antiserum was conjugated with fluorescein isothiocyanate (FITC) (Polyclonal antibody, DAKO A/S, Denmark) that was diluted to 1 : 40 and applied to fixed cells and incubated for 30 min at 37°C. The fixed cells were washed and counterstained with Evans blue for 5 min, then washed again with distilled water, air dried, and mounted with PBS-glycerol buffer. The direct immunofluorescence of the HSV-infected Vero cells was examined under a fluorescent microscope. The negative control was acetone-fixed uninfected cells which processed the same as the test. The infected cells were observed, scored, and recorded for the fluorescent pattern, localization, and intensity of the fluorescent conjugate of antiserum. 

*Preparation of the Plant Extract, the Herbal, and the Placebo Gels*. The plant was macerated with ethanol and evaporated as dry extract, which was standardized with **7**, as a specific marker compound or standard. The standardized ethanol extract was used as drug material, which was prepared as 1 and 2% herbal gels. Carbopol, the gelling substance, was dispersed in cool boiled water and neutralized with sodium hydroxide solution. The plant extract was dissolved in propylene glycol, added to the gel, and mixed thoroughly. The placebo gel was prepared similarly, but the coloring agent (caramel) was used instead of the plant extract.

*Patients and Method*. This study is a double-blind placebo trial in the treatment of recurrent herpes labialis with *G. procumbens* gel compared to placebo. It was approved by the Ethical Clearance Committee on Human Rights Related to Researches Involving Human Subjects. The clinical trial was performed in accordance with the ICH-GCP guidelines and the Declaration of Helsinki. The patients were informed prior to commencement of the trial and provided written consensus of the participation. It was conducted at three hospitals, that is, Ramathibodi, Chulalongkorn, and Phra Mongkutklao hospitals.

The participated patients were more than 18 years of age, diagnosed as recurrent herpes labialis, and the symptom appeared within 48 hours. The exclusion criteria were the patients during pregnancy or lactation and the patients with chronic diseases and HIV infection. 

On the first day (D0) of the trial commencement, the history and symptoms of the patients were recorded and the infected lesions were photographed. The Tzanck smear and viral culture were performed to confirm the diagnosis. The baseline laboratory examination included CBC, SGOT, SGPT, BUN creatinin, and urine examination.

The patients were allocated into three groups by block randomization. They were group A receiving 1% herbal gel, group B receiving 2% herbal gel, and group C receiving the placebo gel. All patients were supplied with identical tubes of gel. 

They were advised to apply the gel thinly on the infected area every two hours on the first day and four times a day on the following days until the lesion healed. The severity of pain and itch was recorded daily by the patient on a linear visual analogue scale of 0–10 from none to very severe. Dates of full crusting and complete healing were also recorded. The patients were assessed by the investigators on days 2 or 4 and 7 and the day after which lesions completely healed. The assessment on each follow-up visits included the severity of pain and itch and days of full crusting and complete healing of the lesions. The adverse reactions or any patient complaints were also recorded. On D2–D4 and D7, the lesions were photographed and viral cultures were performed. The blood and the urine were collected and examined again on the last visit.

*Statistical Methods*. We calculated that if success rate occurred in 80% of patients with drug treatment and 50% for placebo patients, it would require 45 drug-treated and 45 placebo patients to detect the success rate at 5% significance level with a power of 80%. We inflated our calculated sample size to 50 patients to compensate for 10% of loss follow-up rate. The Fisher exact test was used to compare the success rate between the drug-treated and placebo groups. Descriptive statistics were reported as mean with standard deviation. Statistical analysis was performed on a completed study basis.

## 3. Results

The isolation of aqueous methanol F2 and the methanol F3 fractions resulted in four antiherpetic components, that is, **2** from F2 (Figure 1, Table 1) and **7**, **8**, and **9** from F3 (Figure 2, Table 1). The flavonoids (a mixture of **3.1** and **3.2** and **5**) which were isolated from F2 and **6** from F3 had no antiviral activity. Compounds **2**, **7**, **8**, and **9** were identified using spectroscopic methods, especially NMR with field gradient technique [11–14].

***2***: *A Mixture of Dicaffeoylquinic Acids *(***2.1*** *and* ***2.2***) 

***2.1*** *(Figure 4)*:* 3,5-Di-O-Caffeoylquinic Acid*. Amorphous yellow powder; *R*_f_ 0.55, silica gel 60, CHCl_3_/MeOH/H_2_O/acetic acid/(21 : 15 : 3 : 1); UV (MeOH) *λ*_max⁡_ 326 nm. ^1^H-NMR (CD_3_OD, 300 MHz) *δ* 7.62, 7.58 (d, *J* = 16 Hz, H-7′, H-7′′, each) 7.08, 7.06 (d, *J* = 8.2, H-2′, H-2′′, each) 6.98, 6.96 (dd, *J* = 8, 2 Hz, H-6′, H-6′′, each) 6.78 (d, *J* = 8.0, H-5′, H-5′′, each) 6.41, 6.30 (d, *J* = 16 Hz, H-8′, H-8′′) 5.55 (ddd, *J* = 10.2, 9.7, 6.0, H5ax) 5.38 (ddd, *J* = 3, 3, 3 Hz, H-3eq) 3.90 (dd, *J* = 7, 3 Hz, H-4ax) 2.28 (dd, *J* = 15.1, 3.1 Hz, H-2). 

***2.2*** *(Figure 5)*:* 4,5-Di-O-Caffeoylquinic Acid*. Amorphous yellow powder, *R*_f_ 0.50, silica gel 60, CHCl_3_/MeOH/H_2_O/acetic acid/(21 : 15 : 3 : 1); UV (MeOH) *λ*_max⁡_ 326 nm. ^1^H-NMR (CD_3_OD, 300 MHz) *δ* 7.57, 7.49 (d, *J* = 16 Hz, H-7′, H-7′′, each) 7.02, 6.98 (d, *J* = 2 Hz, H-2′, H-2′′, each) 6.89, 6.86 (dd, *J* = 8, 2 Hz, H-6′, H-6′′, each) 6.73, 6.71 (d, *J* = 8 Hz, H-5′, H-5′′, each) 6.26, 6.18 (d, *J* = 16 Hz, H-8′, H-8′′, each) 5.69 (ddd, *J* = 10.3, 9.7, 7.0 Hz, H-5ax) 5.10 (dd, *J* = 10.0, 3.0 Hz, H-4ax) 4.31 (ddd, *J* = 3.0, 4.0, 3.0 Hz, H-3eq) 2.0–2.4 (broad m, H_2_-2, H_2_-6).

***7***:* A Mixture of *β*-Sitosteryl *(***7.1***,  *Figure 11*) *and Stigmasteryl *(***7.2***,  *Figure 12*) *Glucosides*. White powder; mp 254–256°C (with decomposition); *R*_f_ 0.48, silica gel 60, CHCl_3_/MeOH/H_2_O/(20 : 5 : 0.5); FAB-MS [M-Na]^+^ *m/z* 599 and 597 (calcd for C_35_H_60_O_6_ 576 and C_35_H_58_O_6_ 574 for **7.1** and **7.2**); EI-MS (pos. ion mode) *m/z* 414 and 412 [M+H]^+^ (corresponding aglycones); *m/z* 396 and 394 (low intensity) [M+H-H_2_O]^+^; ^1^H-NMR (C_5_D_5_N, 300 MHz) *δ* 5.35 (dd, *J* = 4.5, 1.5 Hz, H-6) 5.23 (dd, *J* = 15.5, 9 Hz, H-22, **7.1**) 5.08 (dd, *J* = 15.5, 9 Hz, H-23, **7.1**) 5.04 (d, *J* = 7.9 Hz, H-1′) 4.55 (dd, *J* = 11.6, 1.8 Hz, H-6′a) 4.40 (dd, *J* = 11.6, 4.9 Hz, H-6′b) 4.26 (dd, *J* = 15.6, 8.5 Hz, H-3′, H-4′) 4.04 (t, *J* = 8.2, 7.9 Hz, H-2′) 3.96 (m, H-3, H-5′) 2.72–2.47 (m, H-4) 2.14–1.74 (m, H-2) 1.93 (m, H-1) 1.7 (m, H-25) 1.4 (m, H-20) 1.0–1.8 (m, H-8, H-9, H-14, H-17) 1.28 (m, H-28) 1.09 (d, *J* = 6.7 Hz, H_3_-21, **7.2**) 1.0 (d, *J* = 6.4, H_3_-21, **7.2**) 0.94, 0.96 (m, H-24) 0.93 (d, *J* = 6.9 Hz, H_3_-27) 0.92 (d, *J* = 7 Hz, H_3_-26) 0.95 (s, H_3_-19) 0.90–1.30 (m, H-23, H-23, **7.2**) 0.90 (d, *J* = 6.1 Hz, H_3_-27) 0.89 (d, *J* = 6.7 Hz, H_3_-26) 0.88 (t, *J* = 7.67, 7.67 Hz, H_3_-29) 0.8–2.2 (m, H-7, H-11, H-12, H-15, H-16) 0.69 (s, H_3_-18, **7.1**) 0.67 (s, H_3_-18, **7.2**); ^13^C-NMR (C_5_D_5_N, 75 MHz) *δ* 140.955 (C-5) 138.833 (C-22) 129.521 (C-23) 121.921 (C-6) 102.607 (C-1′) 78.605 (C-3, 3′) 78.440 (C-5′) 75.331 (C-2′) 71.761 (C-4′) 62.894 (C 6′a, 6′b) 56.971 (C-14, **7.2**) 56.889 (C-14, **7.1**) 56.297 (C-17, **7.1**) 56.132 (C-17, **7.2**) 51.460 (C-24, **7.1**) 50.404 (C-9) 46.097 (C-24, **7.2**) 42.527 (C-13, **7.1**) 42.396 (C-13, **7.2**) 40.783 (C-20, **7.1**) 40.010 (C-12) 39.368 (C-4) 37.526 (C-1) 36.967 (C-10) 36.424 (C-20, **7.2**) 34.269 (C-22, **7.2**) 32.196 (C-7) 32.114 (C-8, 25 **7.1**) 30.290 (C-2) 29.531 (C-25, **7.2**) 29.317 (C-16, **7.1**) 28.560 (C-16, **7.2**) 25.714 (C-28, **7.1**) 24.579 (C-15, **7.2**) 24.546 (C-15, **7.1**) 23.444 (C-28, **7.1**) 21.502 (C-21, **7.1**) 21.305 (C-11) 19.446 (C-19, **7.2**) 19.216 (C-19, **7.1**) 19.051 (C-21, **7.2**) 12.191 (C-18, **7.1**) 12.010 (C-18, **7.2**).

***8***:  *1,2-bis-Dodecanoyl-3-*α*-D-Glucopyranosyl-sn-Glycerol * (*Monoglucosyl Diglyceride, Figure 13*). Colorless waxy mass, *R*_f_ 0.15, silica gel 60, CHCl_3_/MeOH/H_2_O/(20 : 5 : 0.5); ^1^H-NMR (CD_3_OD), 300 MHz) *δ* 5.28 (dddd, *J* = 7.0, 6.4, 4.0, 4.1 Hz, H-2) 4.77 (d, *J* = 3.8 Hz, H-1′′′) 4.50 (m, H-1a) 4.14 (dd, *J* = 12.0, 7.0 Hz, H-1b) 4.00 (dd, *J* = 10.8, 6.4 Hz, H-3a; ddd, *J* = 9.8, 9.0, 3.4 Hz, H-5′′′) 3.60 (dd, *J* = 9.5, 9.0 Hz, H-3′′′) 3.56 (dd, *J* = 10.8, 6.4 Hz, H-3b) 3.41 (dd, *J* = 9.7, 3.8 Hz, H-2′′′) 3.28 (dd, *J* = 14.3, 9.7 Hz, H-6′′′a) 3.19 (dd, *J* = 9.7, 9.0 Hz, H-4′′′) 3.00 (dd, *J* = 14.3, 3.4 Hz, H-6′′′b) 2.30 (t, *J* = 7.6, 7.6 Hz, H-2′, 2′′) 1.57 (m, H-3′, 3′′) 1.25 (m, H-4′-13′, 4′′-13′′) 0.84 (t, *J* = 6.4, 6.0 Hz, H-14′, 14′′); ^13^C-NMR (CD_3_OD, 75 MHz): *δ* 192.31 (C-1′, 1′′) 99.08 (C-1′′′) 74.00 (C-3′′′, 4′′′) 73.00 (C-2′′′) 71.50 (C-2) 70.00 (C-5′′′) 66.00 (C-3a, 3b) 64.00 (C-1a, 1b) 61.00 (C-6′′′a, 6′′′b) 34.69 (C-2′, 2′′) 29.52–30.06 (C-4′-11′, 4′′-11′′) 25.27 (C-3′, 3′′) 23.02 (C-13′, 13′′).

***9***:* A Mixture of *β*-Sitosterol *(***9.1***, *Figure 14*) *and Stigmasterol *(***9.2***, *Figure 15*). White needles, mp 145–148°C; *R*_f_ 0.75, silica gel 60, CHCl_3_/MeOH/H_2_O/(20 : 5 : 0.5); ^1^H-NMR (CDCl_3_, 300 MHz) *δ* 5.34 (broad, d, *J* = 5.4 Hz, H-6) 5.15 (dd, *J* = 15, 8.5 Hz, H-22, **9.1**) 5.01 (dd, *J* = 15, 8.5 Hz, H-23, **9.1**) 3.52 (dddd, *J* = 11, 10, 5, 4 Hz, H-3) 2.26 (m, H-12) 2.08–1.78 (m, H-22, **9.2**) 1.3–0.8 (m, H-23, **9.2**) 1.7 (m, H-25) 1.28 (m, H-28) 1.2 (d, *J* = 6.4 Hz, H-21) 1–1.18 (m, H-9, 14, 17, 20) 1.0 (s, H_3_-19) 0.95 (m, H-24) 0.84 (d, *J* = 6.4 Hz, H-26*) 0.82 (d, *J* = 6.4 Hz, H-27*) 0.80 (t, *J* = 6.5, 6.5 Hz, H-29) 0.8–2.2 (m, H-1, 2, 7, 11, 15, 16) 0.68 (s, H_3_-18, **9.2**) 0.70 (s, H_3_-18, **9.1**); ^13^C-NMR (CDCl_3_, 75 MHz) *δ* 140.77 (C-5) 138.30 (C-22, **9.1**) 129.30 (C-23, **9.1**) 121.70 (C-6) 71.82 (C-3) 56.88 (C-14, **9.1**) 56.79 (C-14, **9.2**) 56.09 (C-17, **9.2**) 55.99 (C-17, **9.1**) 51.24 (C-24, **9.1**) 50.19 (C-9, **9.1**) 50.16 (C-9, **9.2**) 45.87 (C-24, **9.2**) 42.33 (C-4) 42.23 (C-13) 40.47 (C-20) 39.80 (C-12, **9.2**) 39.70 (C-12, **9.1**) 36.53 (C-10, **9.1**) 36.52 (C-10, **9.2**) 36.15 (C-20, **9.2**) 33.97 (C-22, **9.2**) 32.27 (C-1) 31.92 (C-7, **9.1**) 31.88 (C-7, **9.2**) 31.69 (C-2) 29.19 (C-25, **9.2**) 28.91 (C-16, **9.1**) 28.24 (C-16, **9.2**) 26.13 (C-23, **9.2**) 25.40 (C-28, **9.1**) 24.37 (C-15, **9.1**) 24.31 (C-15, **9.2**) 23.09 (C-28, **9.2**) 21.21 (C-26*, **9.1**) 21.09 (C-21, **9.1**) 19.81 (C-26*, **9.2**) 19.39 (C-19) 19.05 (C-27**, **9.1**) 18.99 (C-27**, **9.2**) 18.79 (C-21, **9.2**) 12.23 (C-29) 12.05 (C-18, **9.1**) 11.86 (C-18, **9.2**). ^∗,∗∗^Pairs interchangeable.

The antiherpetic activities of **1** and **4** were not determined because of the minute amount. The compounds **3**, **5**, and **6**were inactive. The chemical structures of **1**, **3**, **4**, **5**, and **6** were identified as follows.

**Figure 3:**
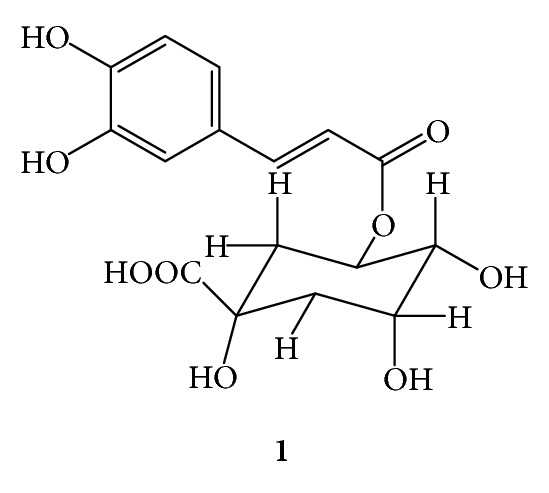


**Figure 4:**
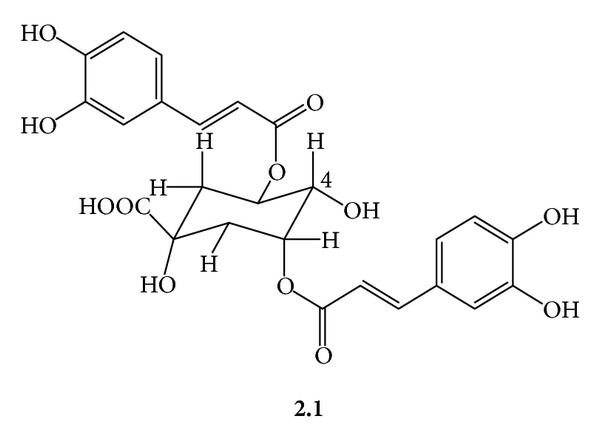


**Figure 5:**
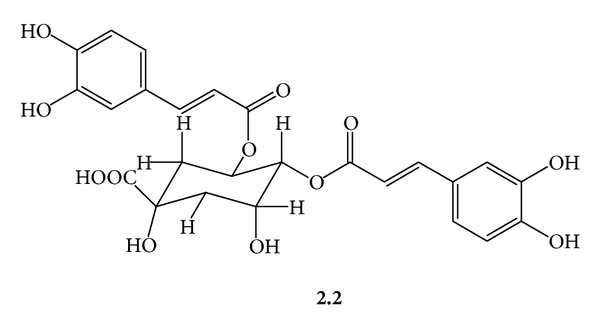


***1***:* 5-O-Caffeoyl-D-Quinic Acid *(*Chlorogenic Acid*,*Figure 3*). Colorless amorphous powder; UV (MeOH) *λ*_max⁡_ 326 nm, shoulder at 299 nm; ESI MS (pos. ion mode) *m/z* 355 [M+H]^+^; (calcd for C_16_H_18_O_9_ 354); ^1^H-NMR (DMSO-*d*_6_, 300 MHz) *δ* 7.40 (d, *J* = 16.0 Hz, H-7′), 7.00 (d, *J* = 2.0 Hz, H-2′), 6.94 (dd, *J* = 8.0, 2.0 Hz, H-6′), 6.73 (d, *J* = 8.0 Hz, H-5′), 6.14 (d, *J* = 16.0 Hz, H-8′), 5.10 (ddd, *J* = 8.0, 7.0, 6.0 Hz, H-5ax), 3.90 (broad, H-3eq), 3.48 (dd, *J* = 7.0, 4.0 Hz, H-4ax), 1.97 (m, H-2a), 1.86 (m, H-6), 1.71 (m, H-2b); ^13^C-NMR (DMSO-*d*_6_, 75 MHz): *δ* 175.00 (C-7), 165.90 (C-9′), 148.20 (C-3′), 145.47 (C-4′), 144.67 (C-7′), 125.56 (C-1′), 121.18 (C-6′), 115.68 (C-5′), 114.70 (C-8′), 114.45 (C-2′), 73.20 (C-1), 71.50 (C-3), 71.19 (C-5), 69.52 (C-4), 36.92 (C-2).

**Figure 6:**
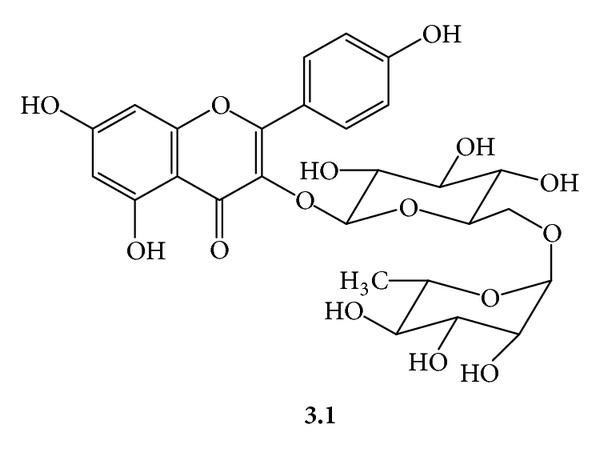


**Figure 7:**
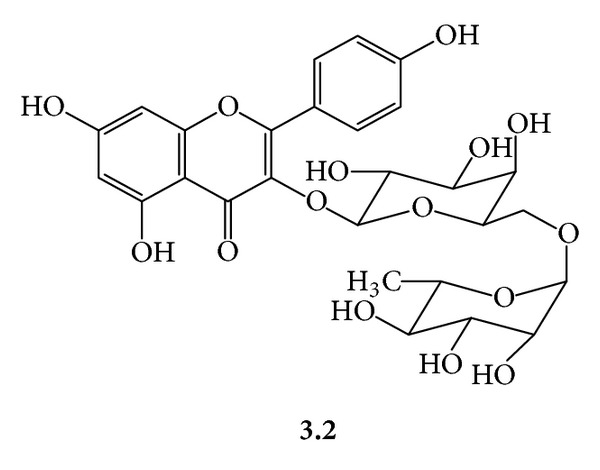


***3***:* A Mixture of Kaempferyl-3-O-*α*-L-Rhamnosyl *(*1→6*)*-*β*-D-Glucopyranoside *(***3.1***,*Figure 6*)* and Kaempferyl-3-O-*α*-L-Rhamnosyl *(*1→6*)*-*β*-D-Galactopyranoside* (***3.2***,*Figure 7*). Yellow amorphous powder, mp 188–190°C (Lit. mp 198–200°C) [12]; UV (MeOH) *λ*_max⁡_ 265 nm (band II) and 349 nm (band I); ^1^H-NMR (DMSO-*d*_6_, 300 MHz) *δ* 12.48 (d, *J* = 3.7 Hz, 2 × 5-OH) 10.10 (OH) 8.04 (d, *J* = 8.9 Hz, H-2′, H-6′, **3.1**) 7.96 (d, *J* = 9.0 Hz, H-2′, H-6′, **3.2**) 6.87 (d, *J* = 8.9 Hz, H-3′, H-5′, **3.1**) 6.85 (d, *J* = 8.9 Hz, H-3′, H-5′, **3.2**) 6.18 (d, *J* = 2.0 Hz, H-6a, H-8a) 6.40 (d, *J* = 1.8 Hz, H-6b) 6.38 (d, *J* = 2.0 Hz, H-8b) 5.31 (d, *J* = 7.5 Hz, H-1′′) 5.27 (d, *J* = 1.5 Hz, H-1′′) 5.16 (d, *J* = 4.4 Hz, OH) 5.05 (d, *J* = 4.7 Hz, OH) 5.03 (d, *J* = 6.3 Hz, OH) 4.88 (d, *J* = 5.0 Hz, OH) 4.55 (d, *J* = 5.3 Hz, OH) 4.52 (d, *J* = 4.1 Hz, OH) 4.38 (d, *J* = 6.0 Hz, H-1′′′) 4.37 (d, *J* = 6.0 Hz, H-1′′′) 3.68 (d, *J* = 10.0 Hz, OH) 3.58 (m, H-2′′, H-4′′, H-6′′) 3.10–3.58 (m, H-3′′, H-5′′, H-2′′′, H-3′′′, H4′′′, H-5′′′) 1.05 (d, *J* = 6.1 Hz, 6′′′-CH_3_-Rha) 0.97 (d, *J* = 6.3 Hz, 6′′′-CH_3_-Rha). 

^13^C-NMR (DMSO-*d*_6_, 75 MHz) *δ* 177.00 (C-4) 165.00 (C-7) 161.00 (C-5) 160.00 (C-4′) 156.00 (C-2, C-9) 133.00 (C-3) 131.00 (C-2′, C-6′) 121.00 (C-1′) 115.01 (C-3′, C-5′, **3.2**) 115.00 (C-3′, C-5′, **3.1**) 103.00 (C-10) 101.00 (C-1′′) 100.00 (C-1′′′) 98.50 (C-6) 93.50 (C-8) 75.65 (C-3′′, **3.1**) 75.50 (C-5′′, **3.1**) 73.93 (C-2′′, **3.1**) 73.31 (C-5′′, **3.2**) 72.74 (C-3′′, **3.2**) 71.50 (C-2′′, **3.2**) 71.50 (C-2′′′, C-4′′′, **3.1**) 70.33 (C-2′′′, C-4′′′, **3.2**) 70.33 (C-3′′′) 70.02 (C-4′′, **3.1**) 67.66 (C-4′′, **3.2**) 67.66 (C-5′′′) 66.49 (C-6′′) 17.41 (C-6′′′, **3.2**) 17.31 (C-6′′′, **3.1**) [13].

**Figure 8:**
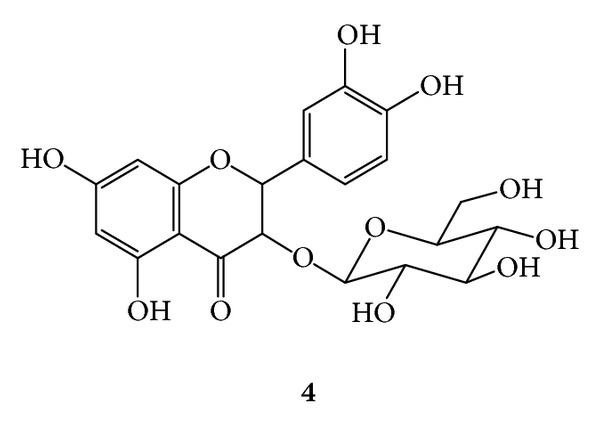


***4***:* Quercetin-3-O-*β*-D-Glucopyranoside (Figure 8)*. Yellow amorphous powder; UV (MeOH) *λ*_max⁡_ 256 nm (band II) and 358 nm (band I); ^1^H-NMR (CD_3_OD, 300 MHz) *δ* 7.72 (d, *J* = 2.1 Hz, H-2′) 7.52 (dd, *J* = 8.5, 2.2 Hz, H-6′) 6.89 (d, *J* = 8.5 Hz, H-5′) 6.36 (d, *J =* 2.0 Hz, H-8) 6.21 (d, *J* = 2.0 Hz, H-6) 5.15 (d, *J* = 7.5 Hz, H-1′′) 3.71 (dd, *J* = 11.9, 2.4 Hz, H-6′′a) 3.56 (dd, *J* = 11.9, 5.2 Hz, H-6′′b) 3.48 (dd, *J* = 8.0, 7.5 Hz, H-2′′) 3.42 (dd, *J* = 9.0, 8.0 Hz, H-3′′) 3.34 (dd, *J* = 10.0, 9.0 Hz, H-4′′) 3.22 (ddd, *J* = 10.5, 5.5, 2.5 Hz, H-5′′); ^13^C-NMR (CD_3_OD, 75 MHz): *δ* 179.49 (C-4) 166.19 (C-7) 159.04 (C-3) 158.49 (C-2, C-9) 149.86 (C-4′) 145.92 (C-3′) 135.60 (C-3) 123.19 (C-1′) 123.09 (C-6′) 117.56 (C-5′) 116.03 (C-2′) 105.39 (C-10) 104.31 (C-1′′) 99.65 (C-6) 94.78 (C-8) 78.38 (C-3′′) 78.11 (C-5′′) 75.72 (C-2′′) 71.24 (C-4′′) 62.56 (C-6′′).

**Figure 9:**
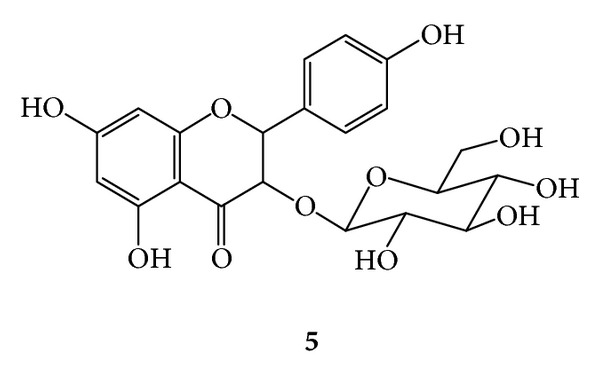


***5***:* Kaempferyl Glucopyranoside (Figure 9)*. Yellow amorphous powder, mp 165–170°C (with decomposition) [14]; UV (MeOH) *λ*_max⁡_ 266 nm (band II) and 349 nm (band I); IR (KBr) *ν*_max⁡_ 3350–3450 (O–H), 2933 (aliph. (C–H)), 1633 (C=O), 1611 (C=C), 1604 (C=O) cm^−1^; ESI MS: *M*_R_ 448. ^1^H-NMR (DMSO-*d*_6_, 300 MHz) *δ* 12.50 (broad s, 5-OH), 8.01 (d, *J* = 9.0 Hz, H-2′, H-6′, each), 6.84 (d, *J* = 9.0 Hz, H-3′, H-5′, each), 6.38 (d, *J* = 2 Hz, H-8), 6.17 (d, *J* = 2 Hz, H-6), 5.41 (d, *J* = 7.1 Hz, H-1′′), 5.24 (2′′-OH), 4.96 (5′′-OH), 4.86 (3′′-OH, 4′′-OH), 4.16 (6′′-OH), 3.55–3.30 (broad s, H-6′′), 3.18 (H-5′′), 3.16 (broad s, H-2′′), 3.06 (broad s, H-3′′, H-4′′); ^13^C-NMR (DMSO-*d*_6_, 75 MHz) 177.13 (C-4), 165.10 (C-7), 161.09 (C-5), 156.43 (C-9), 155.88 (C-2), 133.08 (C-3), 130.75 (C-2′, C-6′), 120.88 (C-1′), 115.02 (C-3′, C-5′), 103.43 (C-10), 100.97 (D, C-1′′), 99.00 (C-6), 93.78 (C-8), 77.41 (C-3′′), 76.39 (C-5′′), 74.17 (C-2′′), 69.84 (C-4′′), 60.80 (C-6′′).

**Figure 10:**
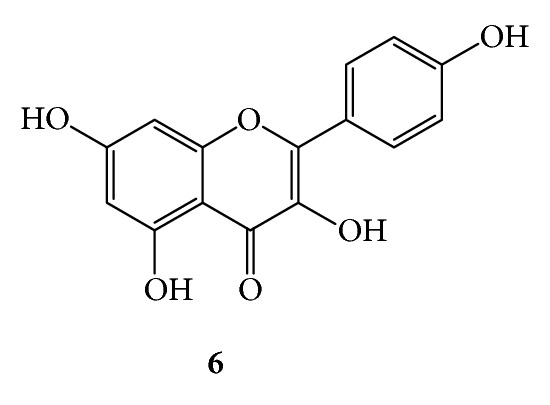


**Figure 11:**
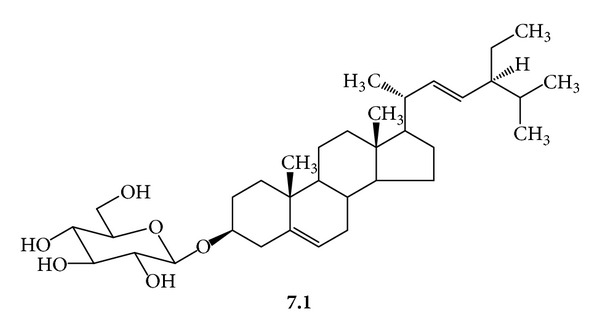


**Figure 12:**
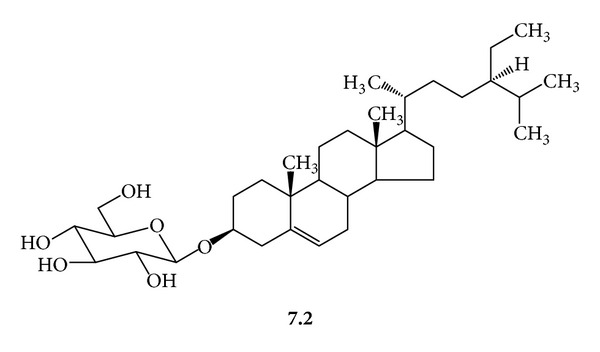


**Figure 13:**
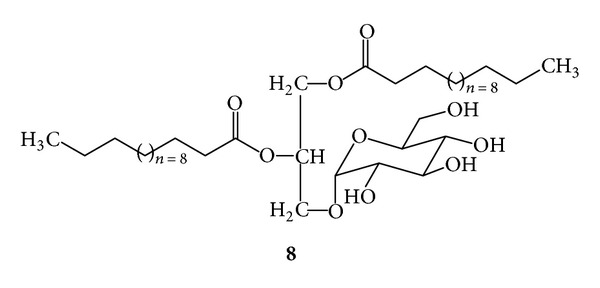


**Figure 14:**
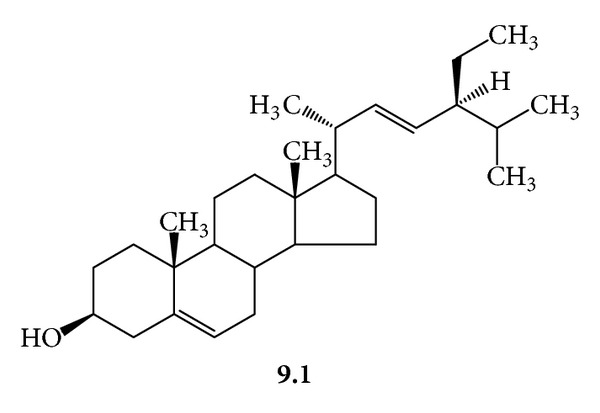


**Figure 15:**
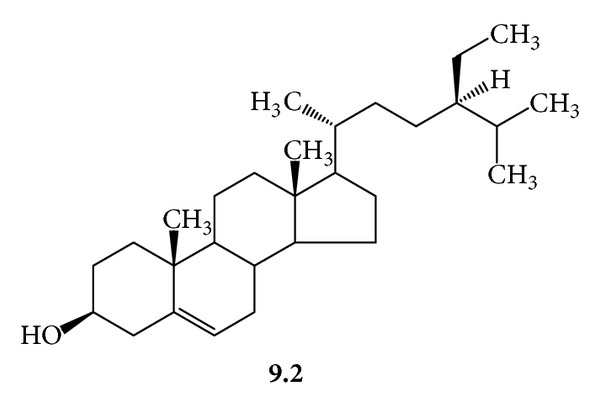


***6***:* Kaempferol *(*3,5,7,4*′*-tetrahydroxyflavone*,*Figure 10*). Yellow amorphous powder; UV (MeOH) *λ*_max⁡_ 260 nm (shoulder) and 365 nm (band I); ^1^H-NMR (CD_3_OD 300 MHz) *δ* 8.07 (d, *J* = 8.5 Hz, H-2′, H-6′), 6.91 (d, *J* = 8.5 Hz, H-3′, H-5′), 6.40 (d, *J* = 1.7 Hz, H-8), 6.19 (d, *J* = 1.7 Hz, H-6); ^13^C-NMR (CD_3_OD, 75 MHz) *δ* 177.42 (C-4), 165.56 (C-7), 162.46 (C-5), 160.56 (C-4′), 158.24 (C-9), 148.10 (C-2), 137.16 (C-3), 130.68 (C-2′, C-6′), 123.76 (C-1′), 116.31 (C-3′, C-5′), 104.56 (C-10), 99.29 (C-6), 94.486 (C-8).

To evaluate the antiherpetic effectiveness of *G. procumbens,* we performed the double-blind, randomized, controlled clinical trial in patients with recurrent herpes labialis of the herbal products, which contained 1 and 2% of the standardized plant extracts.

A total of 65 patients participated in the study. Four patients (three in placebo group, one in the treated group) did not appear for the followup. One patient in the treated group did not record the date of full crusting and complete healing. One patient in the placebo group could not continue treatment due to allergic contact dermatitis. Therefore, only 59 patients were evaluated. They were divided into the treated group with 1% herbal gel (19 patients), the treated group with 2% herbal gel (22 patients), and the placebo group (18 patients). The antiherpetic results of the treated groups (1 and 2% herbal gels) were not different significantly. The data from both treated groups were thus combined (41 patients, 10 males and 31 females) and compared to the placebo group which comprised 18 patients (3 males, 15 females). Both groups were comparable for age, sex, pain, and itching scores, proportion of patient who are suffering from pain, and percentage of patients who had positive tests for Tzanck smear and viral culture. The only significant difference was the proportion of itching patients. It was greater in the treated group as shown in Table 2.

The positive results of Tzanck smear of the treated and control groups were 70.0 and 72.2%, respectively. The viral culture showed the presence of HSV-1 in the treated and control groups 46.2 and 31.3%, respectively. The patients in the treated and control groups were not significantly different, apart from the itching in the treated group, which was significantly different from the control (Table 2).

The first followup (D2, D4) indicated that the treated group still suffered the pain, but the number decreased from 27 patients (65.90%) to 20 patients (34.15%), the itching decreasing from 68.29 to 48.78%. The patients who are suffering from pain in the placebo group decreased from 11 in 18 patients (61.10%) to 6 in 18 patients (33.33%), but the proportion of patients with itching remain unchanged (7 in 18 patients, 38.89%). The average pain scores of the treated and placebo groups were 1.11 and 1.41, respectively. The average itching scores of the treated and the placebo groups were 1.60 and 1.44, respectively. The results of both groups were not significantly different (Table 3).

Twenty-one of 41 patients in the treated group (51.22%) had full crusting within 4 days. It was comparable to 9 of 18 patients (50%) in the placebo group. Lesions completely healed within 7 days in twenty-six patients (63.41%) of the treated group whereas it was 8 in 18 patients (44.44%) for the placebo group. In the treated group, the average time for full crusting was 4.9 days and for complete healing was 8.7 days. The same figures for the placebo group were 5.1 and 9.4 days, respectively. The viral culture on D7 showed the number of patients in the treated group who had positive culture to be reduced from 19 to 3 whereas those in the placebo group reduced from 5 to 3 patients. The results differed from the placebo insignificantly (Table 3).

*Side Effects*. The blood chemistry (CBC, UA, SGOT, SGPT, BUN, and Cr) of the participated patients was normal after the treatment. There was one patient in the placebo group suffering from allergic contact dermatitis, so the treatment was discontinued. There were 10 patients (24.4%) in the treated group and 5 patients (27.8%) in the placebo group who had mild itching and irritation, but all patients could tolerate the symptoms without disruption of the treatment. The side effects in the treated and placebo groups were not significantly different.

## 4. Discussion

The phytochemical work on *G. procumbens* showed that the ethanol extract of the aerial plant parts, the drug material, had the virucidal action against HSV-1 and HSV-2 (IC_50_ 625.0 and 675.0 *μ*g/mL, resp.) and prevented the viral replication with IC_50_ 584.0 and 568.0 *μ*g/mL, respectively. Several antiherpetic compounds were isolated from the extract. Some of them such as dicaffeoylquinic acids (**2**) were known for the antiviral activity [15]. We found that the extract contained other antiherpetic compounds, that is, **7**, **8**, and **9**. The antiherpetic activity against HSV-2 of **8**, the glycosphingolipid, was stronger than **2** (dicaffeoylquinic acids). In addition, several flavonoids were found in the extract. They probably exerted the activity at the concentration above the MNTD. The test concentrations should not exceed the MNTD because the cultured cells would be damaged. However, the presence of the flavonoids possibly imparted anti-inflammatory effect [1, 4, 16], which could alleviate the infectious symptoms. 

The laboratory evidence directed us to carry on the clinical study. The double-blind randomized, controlled clinical trial of recurrent herpes labialis with herbal and placebo gels was conducted. The ethanol extract was standardized using a mixture of sitosteryl and stigmasteryl glucosides as a marker compound (standard) because it could be isolated in a sufficient amount without difficulty. The standardized extract was incorporated in gel base as 1 and 2% herbal gels. The placebo gel was prepared similarly, colored like herbal gel but without the extract. 

We could not achieve the number of the participated patients as calculated (50 in each group), though the trials were conducted at three hospitals. There were only 65 patients participating in the trial, but only 59 patients completed the study. Thus, the power of this clinical trial reduced from 80% to only 50%. Though the antiherpetic result of herbal gel differed insignificantly from the placebo, it tended to have some benefits. These included the tendency of the decreasing number of patients who suffered from itches in the treated group, the greater number of patients healing within 7 days, and the lesser number of patients infected with HSV-1. From the traditional, laboratory, and clinical pieces of evidence, we could postulate that *G. procumbens* tended to reduce the suffering from HSV-1 infection and might have some antiherpetic action. We performed the stability test of the herbal gel and found that the appearance and the content of the marker compound remained unchanged when storing the gel at 25°C for 24 months and at 40°C for 6 months. To improve the efficacy result of this herbal gel for the treatment of recurrent herpes labialis, we recommend the change of gel base to reduce the irritation and increase the potency of it. For further clinical investigation, we recommend increasing patients number and high concentration of the plant extract in the herbal gel.

## 5. Conclusion

*Gynura procumbens* comprised antiherpetic compounds, that is, caffeoylquinic acid derivatives, phytosteryl glucosides, and glycoglycerolipids. The flavonoids in this plant possibly imparted anti-inflammatory effect which was advantageous to the herpetic patients. The laboratory evidence and the reduction of the infection incidence in patients supported the antiherpetic effect of *G. procumbens*. The insignificant result of the clinical study might arise from the low participated patient number and insufficient extract concentration in the herbal product.

## Figures and Tables

**Table 1:** Antiviral activity of the extract, extract fractions, and isolated compounds from *G. procumbens*.

Extract/extract fractions/compounds	MNTD *μ*g/mL	Antiviral activity (IC_50_, *μ*g/mL)					
		Inactivation (virucidal action)		Pre-treatment inhibition of viral adsorption and penetration		Post-treatment inhibition of intracellular viral replication	
		HSV-1	HSV-2	HSV-1	HSV-2	HSV-1	HSV-2
Ethanol extract	>2000	625	675	−	−	584	568
F2	1000	320	366	−	−	−	−
F3	1000	362	391	−	−	ND	266
F4	1000	347	312	+	+	+	446
1	ND	ND	ND	ND	ND	ND	ND
2	200	+	96	−	−	+	61
3	200	−	−	−	−	−	−
4	ND	ND	ND	ND	ND	ND	ND
5	200	−	−	−	−	−	−
6	10	−	−	−	−	−	−
7	100	+	50	+	+	−	−
8	100	−	40	−	−	−	−
9	500	250	−	ND	ND	−	−

Positive control: 50 *μ*g/mL of Acyclovir completely inhibited the plaque formation in all test methods.

−: inactive at subtoxic concentration (MNTD/2) (inhibition of plaque forming < 50%).

+: active but IC_50_ is not determined.

MNTD: maximum nontoxic dose.

ND: not determined.

**Table 2:** Patients clinical and laboratory data before treatment in the treatment and placebo groups.

Patients	The treated groups	The placebo groups	*P* value
Sex			
Male	10	3	0.51
Female	31	15	
Total	41	18	
Age (years)			
Average	37.15	41.72	
Range	16–71	20–69	0.20
Standard deviation	11.31	15.25	
Pain on the first day (D0)			
Case/total (%)	27/41 (65.90%)	11/18 (61.10%)	0.72
Pain score (average)	2.85	2.86	0.90
Itching on the first day (D0)			
Case/total (%)	28/41 (68.29%)	7/18 (38.89%)	0.03
Itching score (average)	2.73	2.10	0.46
Tzanck smear before the treatment			
Positive result (%)	70.0	72.2	0.86
Viral culture before the treatment			
Positive result (%)	19/39 (48.7)	5/16 (31.25)	0.23

**Table 3:** The results of the double-blind randomized, controlled clinical trial of *Gynura procumbens* gel product.

Result	The treated groups	The placebo groups	*P* value
The pain on D2–D4			
Number of patients/total (%)	14/41 (34.15)	6/18 (33.33)	0.95
Average pain score	1.11	1.41	0.65
The itching from D2–D4			
Number of patients/total (%)	20/41 (48.78)	7/18 (38.89)	0.48
Average itching score	1.60	1.44	0.80
The crust day			
Within 4 days (patients/total)	21/41 (51.22%)	9/18 (50.00%)	0.93
Average time (days)	4.9	5.1	0.84
The healing day			
Within 7 days (patients/total)	26/41 (63.41%)	8/18 (44.44%)	0.18
Average time (days)	8.7	9.4	0.56
The viral culture			
Proportion +ve culture (%)			
On D2–D4	8/38 (21.05)	3/15 (20.0)	1.0
On D7	3/39 (7.69)	3/15 (20.0)	0.33
